# Association of lumbar vertebral hemangiomas with low back pain, morphological changes, and quality of life: a cross-sectional study

**DOI:** 10.1186/s12891-026-10021-w

**Published:** 2026-06-02

**Authors:** Daishui Yang, Luis Becker, Lukas Mödl, Tianwei Zhang, Sihai Liu, Bernhard U Hoehl, Matthias Pumberger, Hendrik Schmidt

**Affiliations:** 1 Julius Wolff Institute, Berlin Institute of Health at Charité - Universitätsmedizin Berlin, Augustenburger Platz 1, Berlin, 13353 Germany; 2 Centrum für Muskuloskeletale Chirurgie, Charité - Universitätsmedizin Berlin, Charitéplatz 1, Berlin, 10117 Germany; 3 Institute of Biometry and Clinical Epidemiology, Charité - Universitätsmedizin Berlin, Charitéplatz 1, Berlin, 10117 Germany

**Keywords:** Lumbar Vertebral hemangiomas, Intermitted low back pain, Chronic low back pain, Magnetic resonance imaging, Morphological changes, Disc degeneration

## Abstract

**Background and objective:**

Vertebral hemangiomas are common benign spinal lesions. But studies of lumbar vertebral hemangiomas (LVHs) are still relatively limited. In this work, we assessed the distribution of LVHs within the study population, and explored their relationships with different categories of low back pain (LBP), pain- and health-related outcomes, and lumbar spine morphology.

**Methods:**

A retrospective cross-sectional study recruited 1,021 participants with or without LBP. Magnetic resonance imaging was used to assess the location, size as well as types of LVHs. Clinical data were collected from well-designed questionnaires and clinical examination.

**Results:**

LVHs were identified in 8.3% of the recruited participants, and their prevalence increased with advancing age. The prevalence of LVHs differed across back pian categories, with rates of 12.4% in intermittent LBP groups, 10.0% in chronic LBP group, and 6.3% in the no back pain group. No statistically significant associations were observed between LVHs and either intermittent LBP (OR 1.96, *p* = 0.061) or chronic LBP (OR 1.40, *p* = 0.312). Additionally, pain intensity (*p* = 0.401), functional impairment (*p* = 0.110), and quality of life (*p* = 0.670) did not differ significantly between the no-LVHs and LVHs groups. Across different LBP categories, no significant difference in LVHs size, type, or number were observed among participants. Following age- and sex-adjusted propensity score matching, greater lumbar disc degeneration (*p* = 0.004) and a trend toward a higher prevalence of Modic changes (*p* = 0.060) were observed in participants with LVHs. No significant differences were found in facet joint degeneration, disc herniation, spinal canal width, or Schmorl’s nodes between the LVHs and matched groups.

**Conclusion:**

In our study, LVHs were not associated with differences in LBP categories or health-related indicators. Additionally, participants with LVHs showed more severe lumbar disc degeneration but no other morphological abnormalities.

**Level of evidence:**

Level 3.

**Supplementary Information:**

The online version contains supplementary material available at 10.1186/s12891-026-10021-w.

## Introduction

Vertebral hemangiomas (VHs) are common benign spinal lesions, found in approximately 11–26% of the general population [1, 2]. The lumbar vertebrae, following the thoracic vertebrae, are the second most frequently affected sites of hemangiomas. Although VHs can affect individuals of all ages, including children, they are more commonly observed in middle-aged and elderly populations [1]. Low back pain (LBP) affects approximately two-thirds of individuals worldwide and negatively impacts patients’ daily lives and work productivity [3]. However, systematic research on the association between lumbar vertebral hemangiomas (LVHs) and LBP remains limited.

Hemangiomas consist of disordered blood vessels that can replace the bone marrow of the vertebrae [2]. According to previous research, VHs fall into three distinct subtypes based on radiology: typical, atypical, and aggressive. Among these, typical VHs are the most frequently encountered type [4]. VHs can potentially disrupt local vascular perfusion in the involved vertebrae and adjacent soft tissues, which can lead to tissue adaptations at the vertebral body and adjacent intervertebral discs [5]. As previous research has reported, some hemangiomas are likely to cause musculoskeletal conditions, such as degenerative diseases and pathological fractures [6, 7]. It remains unclear whether hemangiomas are associated with other adverse changes in the lumbar spine, such as enhanced lumbar disc degeneration (LDD) and facet joint degeneration (FJD). Studies have reported that hemangiomas may extend into the paravertebral space and, consequently, compress the posterior spinal canal [8, 9]. However, the status of surrounding soft tissues in the lumbar spine remains insufficiently explored.

Therefore, our study aimed to (1) assess the association between common demographic characteristics and LVHs, (2) systematically analyze the association between LVHs and LBP prevalence as well as pain- and health-related outcomes, and (3) explore the association between the prevalence of LVHs and lumbar spine morphometric changes. It was hypothesized that (1) individuals with LVHs have a higher occurrence of chronic LBP (cLBP) and intermittent LBP (iLBP), (2) the size of LVHs is significantly associated with cLBP and iLBP, and (3) individuals with LVHs exhibit advanced LDD.

## Materials and methods

### Study population

This was a retrospective, community-based cross-sectional study. MRI data and demographics were collected as part of the ongoing ‘Berlin Back Study’ which aims to develop new strategies to enhance the diagnosis of LBP. The results were reported in accordance with the STROBE guidelines. Participants with or without LBP, recruited between January 2022 and January 2024, were included. The inclusion criteria were patients aged ≥ 18 years. Exclusion criteria included professional athletes, pregnancy, neurological impairments, vertebral fractures, severe cardiovascular disease, or any other reason preventing the individual from undergoing MRI evaluation. A self-reported questionnaire was used to obtain common demographic data, including age, sex, body mass index (BMI, kg/m^2), smoking status, and alcohol frequency **(**Supplementary Questionnaire 1).

### Clinical assessment

Back symptoms were obtained via a self-reported questionnaire and clinical interview, which collected individuals’ back pain status, pain location, and duration (Supplementary Questionnaire 2). Subsequently, the orthopedic physician inquired about the participants’ medical history and conducted physical examination of the spine. Combining the collected data from questionnaires and clinical examinations, our team divided the recruited population into four groups: never had back pain before (no-BP), experienced chronic LBP (cLBP), experienced intermittent LBP (iLBP), and others (including back pain located in the thoracic segment, back pain occurring in the past, and other unidentified types). The present study focused only on the non-BP, cLBP, and iLBP groups. The Short Form-36 (SF-36; 0–100) was used to assess life quality across all populations [10]. Numerical rating scale (NRS; 0 ~ 10) and Roland-Morris disability questionnaire (RMDQ; 0 ~ 24) were used to evaluate the pain intensity and functional impairment in the LBP populations, respectively [11, 12].

### Radiographic evaluation

The Siemens Lumina 3T MRI system (Siemens AG, Germany) was used to perform MRI scans, with both axial and sagittal images. The scanning parameters in the sagittal plane were as follows: repetition time of 682 ms in T1 and 4800 ms in T2, echo time of 11 ms at T1 and 70 ms at T2, and slice thickness of 3 mm in both T1 and T2. The scans were performed in a relaxed, supine position. The lumbar MRI scans for all participants included at least the range from T12 to S1.

### Morphometric alterations

**Fig. 1:**
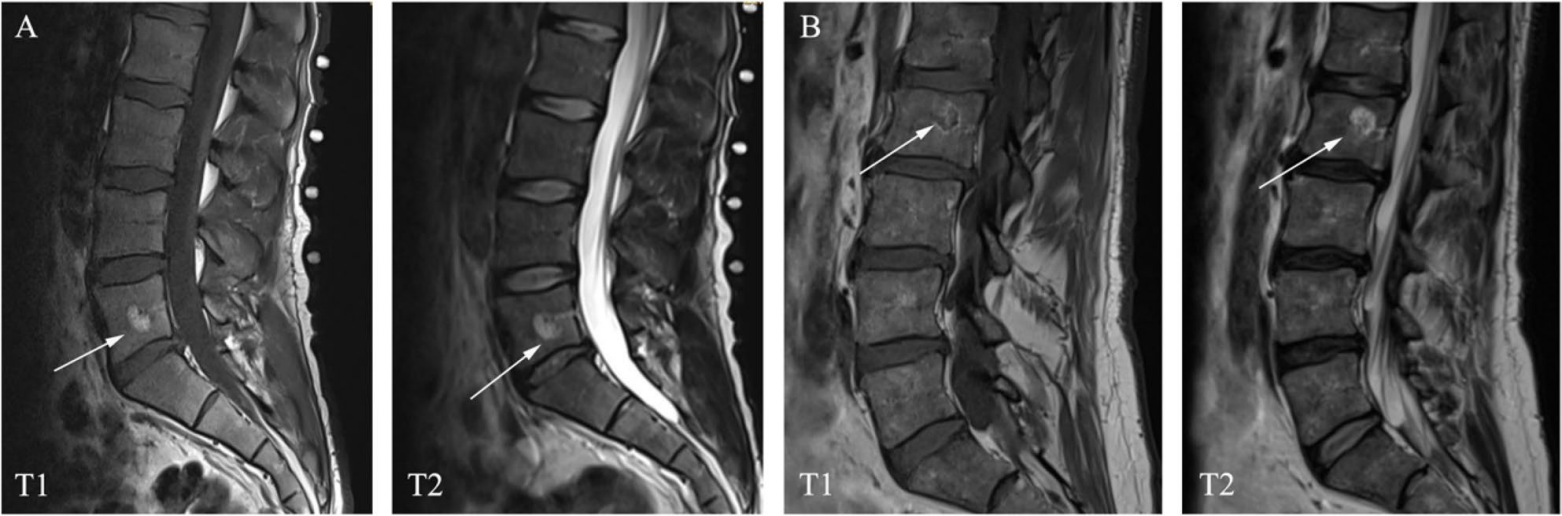
Two distinct types of VHs occurred in the sagittal plane of the lumbar spine. Typical VHs: hyperintense lesion on T1- and T2-weighted images (Figure A); Atypical VHs: hypointense on T1-weighted and hyperintense on T2-weighted (Figure B)

LVHs were evaluated on lumbar MRI based on T1 and T2 weighted image in the sagittal plane. As aggressive LVHs are difficult to diagnose by MRI without contrast medium, in our study, LVHs were divided into two types: typical (tLVHs) and atypical (aLVHs) (Fig. 1). The location, size (including maximum diameter and area in the sagittal plane), and type and number of hemangiomas were measured.

LDD according to the Pfirrmann grading (ranged from 1 ~ 5, higher score indicates more severe degeneration) and lumbar disc herniation (LDH) according to the Krämer classification (range from 0 ~ 5, higher score indicates more severe herniation) were evaluated [13, 14]. FJD (range from 1 ~ 4, higher score signifies more severe herniation) according to Fujiwara classification, Modic changes (MCs, no/yes), spinal canal width (SCW, mm), and Schmorl’s nodes (SNs, no/yes) in the vertebrae were assessed [15, 16].

All morphometric parameters were independently assessed by two experienced physicians. To maintain participant privacy, MRI datasets were de-identified ahead of analysis. The extent of LDD, LDH, FJD, and SCW throughout the entire lumbar spine was assessed by averaging scores from levels L1/2–L5/S1. The presence of MCs or NSs across the entire lumbar spine is indicated by detection at any segments L1/L2–L5/S1.

### Statistical analysis

The Weighted Kappa coefficient (κ) and intraclass correlation coefficient (ICC) were employed to evaluate the consistency of the morphometric alteration data measured by two observers. In addition, to rule out the relevant factors affecting spine morphometric alterations, propensity score matching was performed. Participants with LVHs were matched according to age and sex to controls without LVHs. The nearest-neighbor method was used with a 1:1 matching ratio. The covariate balance was evaluated by calculating the absolute standardized mean difference. Matching was considered acceptable when the value of absolute standardized mean difference was < 0.1 [17]. The chi-square test and Spearman’s rank correlation coefficient were used to compare categorical data, while Dunnett’s test and t-test were used to analyze continuous data. Binary logistic regression was used to assess the association between LBP and LVHs after adjusting for age and sex.

Statistical analyses were performed using Statistical Package for the Social Sciences version 27.0 and R version 4.3.2. The p values were derived from a two-tailed test, with significance defined as *p* < 0.05.

## Results

### Demographic characteristic

1021 participants were recruited for this study (Table 1). Among them, 85 (8.3%) had LVHs. Study participants with identified LVHs had an average age of 49.1 ± 10.1 years and were significantly older than the no-LVHs group (41.6 ± 12.1). The frequency of LVHs was age-dependent and showed significant variation across age cohorts. A prevalence of 3.7% was observed in individuals under 40 years of age, with susceptibility increasing as age advances. Those aged 60 years and above exhibited the highest prevalence of LVHs at 17.5% (*r* = 0.16, *p* < 0.001). The occurrence of LVHs was slightly but not significantly higher among females (9.2%) than among males (7.2%) (*p* = 0.249). Furthermore, the two groups did not differ significantly with respect to BMI (*p* = 0.531), current smoking status (*p* = 0.285), or alcohol consumption frequency (*p =* 0.210).

**Table 1:** Characteristic and demographic between LVHs and no-LVHs groups

Characteristic	LVHs group	no-LVHs group	Prevalence	*P* - value
No. of subject	85	936	8.3%	
Age (median & range, years)	51 (41–57)	41 (31–52)		< 0.001
< 40	17	437	3.7%	< 0.001
40–49	23	207	10.0%	
50–59	31	226	12.1%	
≥ 60	14	66	17.5%	
Sex				
male	32	413	7.2%	0.249
female	53	523	9.2%	
BMI (median & range, kg/m^2)	23.6 (21.5–25.5)	23.9 (22.4–25.3)		0.369
< 25.0	58s	607	8.7%	0.531
≥ 25.0	27	329	7.6%	
Smoke status				
No	73	838	8.0%	0.285
Yes	12	97	11.0%	
Alcohol frequency				
≤ once per week	58	697	7.7%	0.210
> once per week	27	239	10.2%	

Abbreviations:BMI body mass index, LVHs lumbar vertebral hemangiomas

## Distribution and types of LVHs

**Fig. 2:**
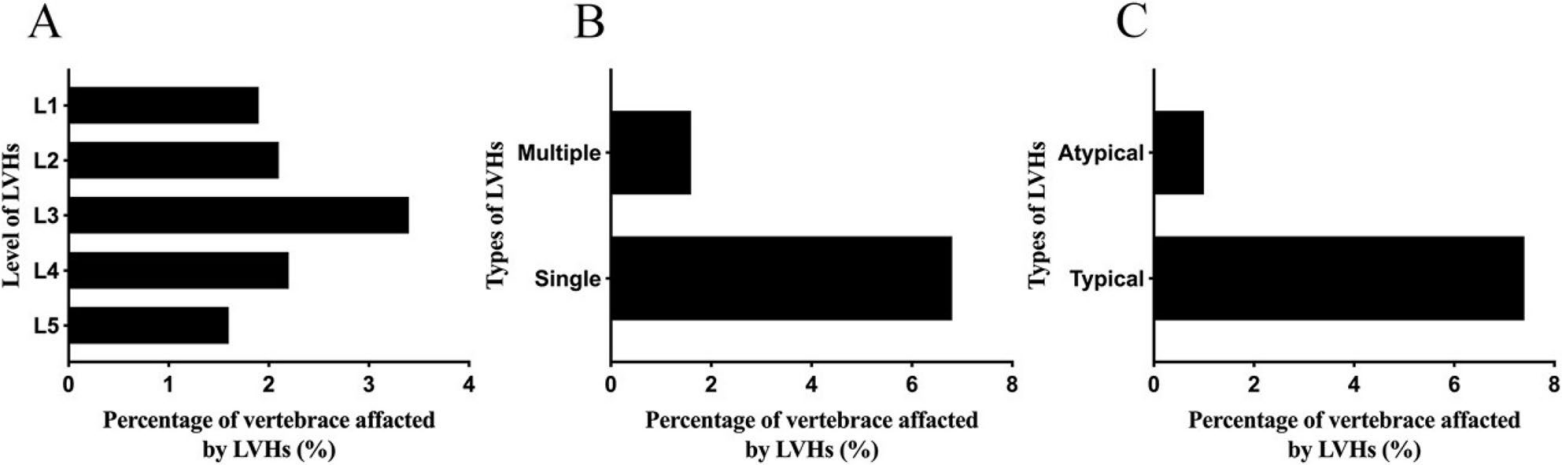
Prevalence of LVHs by location and types. LVHs: lumbar vertebral hemangiomas

The parameters of LVHs and morphometric alterations in the lumbar spine were assessed by two evaluators. All ICC and κ values exceeded 0.60, indicating a strong level of agreement (Supplementary Tables 1 and 2). The most affected vertebra in the lumbar region was L3 (3.4%), followed by L4 (2.2%), L2 (2.1%), L1 (1.9%), and L5 (1.6%) (Fig. 2A). Among the 85 participants with LVHs, 69 (6.8%) had LVHs affecting only one segment, while 16 (1.6%) had LVHs involving at least one additional segment, representing 18.8% of the LVHs group (Fig. 2B). tLVHs were the most observed variety, accounting for 7.3% of the entire cohort, with the remaining aLVHs (1.0%) (Fig. 2C).

### Association with clinical outcomes

**Fig. 3:**
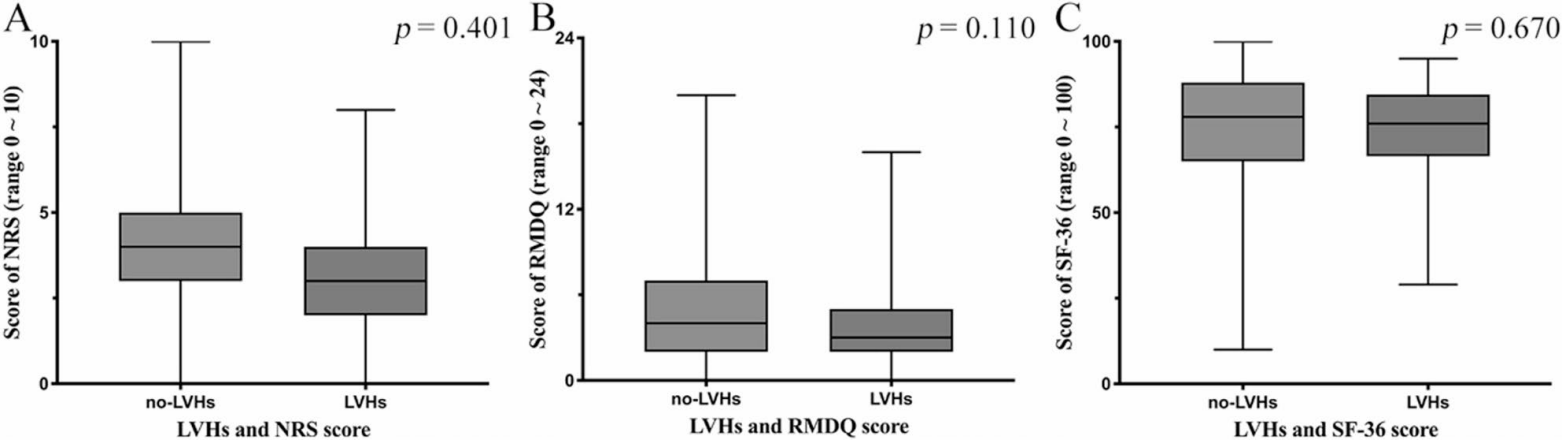
Scores of NRS, RMDQ and SF-36 between on-LVHs and LVHs group. NRS (LBP population): Numerical rating scale, RMDQ (LBP population): Roland-Morris disability questionnaire, SF-36 (entire population): the Short Form-36

The prevalence of LVHs differed across groups, reaching 12.4% in iLBP, 10.0% in cLBP, and 6.3% in the no-BP group (Table 2). Compared with no-LVHs, LVHs individuals showed a higher prevalence of iLBP, but the difference was not significant (OR 1.96, 95%CI 0.97–3.95, *p* = 0.061) and cLBP (OR 1.40, 95%CI, 0.73–2.66; *p* = 0.312) after adjustment for age and sex. Additionally, no differences were found in the scores of NRS (*p* = 0.401), RMDQ (*p* = 0.110), or SF-36 (*p* = 0.670) between the no-LVHs and LVHs groups (Fig. 3).

**Table 2:** The occurrence of LVHs among no-back pain, intermittent LBP and chronic LBP groups

Back pain status	LVHs / no-LVHs	Prevalence	Binary logistic regression (OR, 95%CI; *P* - value)			
			Model 1		Model 2	
no-back pain group	16 / 238	6.3%	1.00 (reference)		1.00 (reference)	
intermittent LBP group	20 / 141	12.4%	2.11 (1.06–4.21)	0.034	1.96 (0.97–3.95)	0.061
chronic LBP group	30 / 270	10.0%	1.65 (0.88–3.11)	0.119	1.40 (0.73–2.66)	0.312

*Abbreviations*: *OR *odds ratio, *CI *confidence interval, *LBP *low back pain.

Model 1: Unadjusted.

Model 2: Adjusted for age and sex (male / female)

Furthermore, there was a broad variability in the size of LVHs, but the means were closely similar in no-BP and cLBP group (16.6 versus 15.6 mm for maximum diameter and 182.8 versus 172.8 mm^2^ for maximum area) (Table 3). The area of LVHs was smaller in the iLBP group than in the non-BP group. There was no difference in the diameter and area of the LVH among the no-BP, iLBP, and cLBP groups (*p* = 0.358 and 0.383, respectively). In addition, the relationship between the type of LVHs and LBP categories were studied. The prevalence of aLVHs gradually increased in the non-BP (6.3%), iLBP (10.0%), and cLBP (16.7%) groups. In addition, most of the LVHs were solitary. The prevalence of multiple LVHs did not differ among the three LBP categories (*p* = 0.480).

**Table 3:** The parameters of LVHs among no-back pain, intermittent LBP and chronic LBP groups

LVHs parameters	no-back pain	intermittent LBP	chronic LBP	*P* - value
	16	20	30	
Diameter (mm)	16.6 (3.1)	15.4 (3.0)	15.6 (2.4)	0.358^a^
LVHs area (mm^2^)	182.8 (58.8)	155.7 (64.6)	172.8 (56.7)	0.383^a^
Types of LVHs, n (%)				
Typical	15 (93.8)	18 (90.0)	25 (83.3)	0.553^b^
Atypical	1 (6.3)	2 (10.0)	5 (16.7)	
Multiple of LVHs, n (%)				
No	12 (75.0)	18 (90.0)	24 (80.0)	0.480^b^
Yes	4 (25.0)	2 (10.0)	6 (20.0)	

*Abbreviations: LVHs *lumbar vertebral hemangiomas, Symbol ‘a’ and ‘b’ indicate that the t-test and chi-square test were conducted

### Association with morphological alterations

After propensity score matching, the matching group included 170 participants: 85 with LVHs and 85 without LVHs (Supplementary Table 3). Participants with LVHs exhibiting greater LDD (Cohen ‘s d = 0.439, *p* = 0.004) and a trend toward a higher prevalence of MCs (Cramer ‘s v = 0.144, *p* = 0.060) (Table 4). In contrast, there was no significant difference in the degree of LDH between the two groups (*p* = 0.405). No association was found between the presence of LVHs and FJD (*p* = 0.662) or SCW (*p* = 0.290). The occurrence rate of SNs was slightly higher in the LVHs group than in the matched group; however, the difference was not statistically significant (*p* = 0.353).

**Table 4:** Morphometric alteration in individuals with and without LVHs groups

Morphometric alterations	LVHs *n* = 85	Matched - group *n* = 85	*P* - value
Intervertebral disc, mean (SD)			
Lumbar disc degeneration	2.3 (0.6)	2.0 (0.7)	0.004^a^
Lumbar disc herniation	0.4 (0.4)	0.5 (0.4)	0.405^a^
Vertebrae body, mean (SD)			
Facet joint degeneration	1.7 (0.4)	1.6 (0.5)	0.662^a^
Spinal canal width (mm)	14.2 (1.5)	14.5 (1.8)	0.290^a^
Modic changes, n (%)			
No	45 (52.9)	57 (67.1)	0.060^b^
Yes	40 (47.1)	28 (32.9)	
Schmorl’s nodes, n (%)			
No	64 (75.3)	69 (81.2)	0.353^b^
Yes	21 (24.7)	16 (18.8)	

*Abbreviations LVHs* lumbar vertebral hemangiomas, Symbol ‘a’ and ‘b’ indicate that the t-test and chi-square test were conducted

## Discussion

This study found a prevalence of 8.3% for LVHs in adults, which is similar to studies based on CT scans (7.0% in the lumbar region) and notably higher than previous reports based on large autopsy series [2, 18]. The reason for this discrepancy could be the improved imaging precision, which makes it possible to identify an increasing number of small vascular tumors. Among common demographic characteristics, age was the only factor linked to LVHs prevalence, consistent with early observation that has shown a low prevalence of LVHs in individuals younger than 40 years and increases significantly among those aged 40 and above [2]. Similar to the majority of earlier studies, there was a slight increase in the incidence of LVHs among females compared to males (with a ratio of 1.44:1) [2, 19, 20]. This difference may be attributed to the higher levels of estrogen in females, which could promote the proliferation of endothelial cells and increase the size of lesions [21]. Nonetheless, no statistically significant difference was found between the sexes in the prevalence of LVHs. This result supports a previous finding that sex hormones are not a decisive factor in causing hemangiomas [22].

It is worth noting that VHs tended to occur in the mid-lumbar spine, with the highest prevalence observed at the L3 level (3.4%). This is consistent with previous studies that reported a higher prevalence at the L1 and L3-4 levels [2]. In our study, the incidence of hemangiomas in the L5 segment (1.6%) and sacral region (< 1%) was low. This may be explained by the hypothesis of Slon et al., which suggests that local pressure may influence the occurrence of hemangiomas. Owing to lordosis of the lumbar spine, the load on the L5 vertebra is distributed through surrounding structures, such as the facet joints and pelvic structure, leading to a comparatively lower pressure on the vertebra itself [2].

Our study is one of the first to systematically explore the association of VHs in the lumbar spine with iLBP and cLBP. LVHs are more prevalent among individuals with iLBP. Additionally, LVHs prevalence appeared higher among individuals with cLBP compared with those without back pain; however, this difference did not reach statistically significance (*p* = 0.159). This may be due to the fact that most of the LVHs observed in our study were typical hemangiomas, whereas previous studies have shown that common typical VHs is often asymptomatic, while aggressive VHs might be accompanied by back pain and in some cases also by myelopathia [1, 23]. Interestingly, compared with the no-BP group, the parameters of LVHs in participants did not differ significantly between the iLBP and cLBP groups, indicating that the size of the lesion was not significantly associated with back pain.

In line with the research conducted by Slon et al., the existence of VHs in the whole spine was positively associated with the development of disc lesions [5]. Our study also revealed a significant increase in the degree of LDD within the LVHs group compared with the matched group. Likewise, when compared with the matched group and the general population in other studies, a slightly higher prevalence of MCs was observed in the LVHs groups [24]. These findings imply that LVHs could potentially disrupt the diffusion of metabolites to the intervertebral discs through the vertebral endplate. Previous studies have suggested that medium-large VHs may influence the blood supply to the vertebrae, which could potentially contribute to the negative effect on vertebral degeneration [5, 6]. In addition, Li et al. reported that individuals with large VHs, comprising more than two-thirds of the vertebral height in the thoracic or lumbar region, are prone to severe pathological fractures [6]. In our study, compared to the matched group, we found that participants with LVH did not show a significant association with FJD and SNs. Our study’s findings may be attributed to the fact that the majority of LVHs were typical hemangiomas and had a moderate diameter, which invaded the marrow cavity and led to the remodeling of bone trabeculae [1].

Our study had some limitations. First, due to the nature of MRI, the size of the LVHs was measured only in the sagittal plane. This may not accurately reflect the true size of the LVHs. Second, the differential diagnosis of atypical vascular tumors based on MRI without a contrast medium remains challenging. It is important to exclude the possibility of metastatic tumors or multiple myeloma, which can mimic the appearance of LVHs on MRI. A comprehensive evaluation that includes clinical symptoms and laboratory data should be considered to aid in accurate diagnosis. Third, due to the community-based study design, the cohort included fewer cases of severe back symptoms or atypical vertebral hemangiomas, limiting wider applicability. Forth, the assessment of lumbar morphometric alterations relies on visual inspection, which could be susceptible to examiner subjectivity. Fifth, as this study was a single-center cross-sectional investigation, selection bias and recall bias may exist in participant recruitment and data collection. Future multicenter, prospective studies should be conducted to enhance the extrapolation of the findings. Finally, we accounted for common confounding variables; however, bias from unmeasured confounding factors may still exist.

## Conclusion

LVHs were more prevalent with increasing age but were not significantly associated with LBP, pain intensity, functional impairment, or quality of life. Furthermore, LVHs seem to be associated with higher degree of LDD. This potential mechanism warrants further investigation.

## Supplementary Information

Supplementary Material 1.

## Data Availability

Data will be made available on reasonable request.

## Abbreviations

LVHs: Lumbar vertebral hemangiomas
tLVHs: Typical LVHs
aLVHs: Atypical LVHs
no-LVHs: No LVHs
LBP: Low back pain
no-BP: No Back pain
iLBP: Intermittent LBP
cLBP: Chronic LBP
LDD: Lumbar disc degeneration
LDH: Lumbar disc herniation
FJD: Facet joint degeneration
SCW: Spine canal width
MCs: Modic changes
SNs: Schmorl’s nodes
SF-36: The Short Form-36
NRS: Numerical rating scale
RMDQ: Roland-Morris disability questionnaire
κ: Weighted Kappa coefficient
ICC: Intraclass correlation coefficient
