# Supplementary material for: Association of lumbar vertebral hemangiomas with low back pain, morphological changes, and quality of life: a cross-sectional study
Source: BMC Musculoskelet Disord. 2026 Jun 2;27:489. doi: 10.1186/s12891-026-10021-w (PMC13242659; doi:10.1186/s12891-026-10021-w)
Supplement: Supplementary file 1 — Supplementary Material 1. [file 12891_2026_10021_MOESM1_ESM.docx]

**Supplementary Material**

**Supplementary Table 1.** Reliability between evaluators with the parameters lumbar vertebrae hemangiomas using intraclass correlation coefficient method

| Parameters | Evaluator 1 vs. Evaluator 2 | |
| --- | --- | --- |
|  | ICC | 95% CI |
| LVHs Diameter (mm) | 0.90 | 0.86 - 0.93 |
| LVHs area (mm^2^) | 0.97 | 0.95 - 0.98 |

ICC Value > 0.8: Very strong agreement. Abbreviations LVHs: Lumbar vertebral hemangiomas, ICC: Intraclass correlation coefficient, CI: Confidence interval

**Supplementary Table 2.** Agreement of measured parameters in MRI imaging by two evaluators

| Parameters | Evaluator 1 vs. Evaluator 2 | |
| --- | --- | --- |
|  | ICC or κ | 95% CI |
| Lumbar disc degeneration | 0.71 | 0.67 - 0.74 |
| Lumbar disc herniation | 0.68 | 0.64 - 0.73 |
| Facet joint degeneration | 0.63 | 0.58 - 0.67 |
| Spinal canal width | 0.92 | 0.91 - 0.93 |
| Endplate degeneration | 0.91 | 0.85 - 0.96 |
| Schmorl’s nodes | 0.78 | 0.66 - 0.90 |

The Weighted Kappa coefficient (κ) method was used for categorical variables, and the intraclass correlation coefficient (ICC) method was conducted for continuous variables. Agreement value ≥ 0.60 was acceptable.

**Supplementary Table 3.** Baseline characteristic of participants before and after propensity score matching

| Characteristic | Before Matching | | | After Matching | | |
| --- | --- | --- | --- | --- | --- | --- |
|  | Control | Hemangioma | ASMD | Control | Hemangioma | ASMD |
| Age | 41.6 (12.1) | 49.1 (10.1) | 0.671 | 49.1 (10.1) | 49.1 (10.1) | <0.001 |
| < 40 | 437 (46.7) | 17 (20.0) | 0.618 | 17 (20.0) | 17 (20.0) | <0.001 |
| 40 - 49 | 207 (22.1) | 23 (27.1) |  | 23 (27.1) | 23 (27.1) |  |
| 50 - 59 | 226 (24.1) | 31 (36.5) |  | 31 (36.5) | 31 (36.5) |  |
| ≥ 60 | 66 (7.1) | 14 (16.5) |  | 14 (16.5) | 14 (16.5) |  |
| Sex |  |  |  |  |  |  |
| male | 413 (44.1) | 32 (37.6) | 0.132 | 32 (37.6) | 32 (37.6) | <0.001 |
| female | 523 (55.9) | 53 (62.4) |  | 53 (62.4) | 53 (62.4) |  |

Means (standard deviations) and numbers (percentages) are presented for continuous and categorical variables, respectively

Abbreviations ASMD: Absolute standardized mean difference

**Supplementary Questionnaire 1**

Pseudonym: ****** Date: yyyy/MM/dd

Age: _____ (years) Gender: _____ (male/female)

Hight: _____ (cm) Weight: _____ (kg)

Do you currently smoke? _____(Yes/No)

How often do you drink alcoholic beverages?

A Never B Less than once a month C Two to four times a month

D Two to three times a week E Four or more times a week

**Supplementary Questionnaire 2**

1. Chronic / intermittent / no back pain? _____(physician-assisted assessment)

2. How long have you had back pain? _____ (years)

3. How would you rate the average intensity of back pain over the past 12 weeks? _____ (Scale: 0–10, with 0 representing no pain and 10 representing the worst possible pain).

4. Where is the primary location of the pain? (with the aid of an illustrative diagram)

A Hip B Pelvis C Sacroiliac joint D Sacrum E Lower lumbar spine

F Middle lumbar spine G Upper lumbar spine H Lower thoracic spine

I Middle thoracic spine J Upper thoracic spine

5. Where is the secondary location of the pain? (with the aid of an illustrative diagram)

A Hip B Pelvis C Sacroiliac joint D Sacrum E Lower lumbar spine

F Middle lumbar spine G Upper lumbar spine H Lower thoracic spine

I Middle thoracic spine J Upper thoracic spine
